# Binding of VEGF-A is sufficient to abrogate the disturbing effects of VEGF-B together with VEGF-A on retinal endothelial cells

**DOI:** 10.1007/s00417-015-2944-z

**Published:** 2015-02-08

**Authors:** Heidrun L. Deissler, Gerhard K. Lang, Gabriele E. Lang

**Affiliations:** Department of Ophthalmology, University of Ulm, Prittwitzstrasse 43, 89075 Ulm, Germany

**Keywords:** Retinal endothelial cells, VEGF-B, Diabetic retinopathy, VEGF-inhibition

## Abstract

**Purpose:**

Inhibition of vascular endothelial growth factor (VEGF) is a promising strategy to treat retinal complications of diabetes. In contrast to VEGF-A binding ranibizumab, aflibercept also binds to other members of the VEGF family including VEGF-B, but potential effects of this factor on permeability and angiogenic processes are unclear. Therefore, we studied how VEGF-B variants as single agents or together with VEGF-A_165_ might affect proliferation, migration, or barrier function of retinal endothelial cells (REC). Also investigated was the normalization of REC properties with both VEGF-inhibitors to explore if additional targeting of VEGF-B is relevant.

**Methods:**

Stimulation of proliferation or migration of immortalized bovine REC (iBREC) and disturbance of their barrier by exposure to VEGF-B variants (as single factors or together with VEGF-A_165_) was determined with or without VEGF-binding proteins being added. Permeability of iBREC was assessed by measuring their transendothelial resistance (TER) and expression of the tight junction protein claudin-1.

**Results:**

VEGF-B_167_ and VEGF-B_186_ enhanced proliferation of iBREC but these isoforms did not affect cell migration. Interestingly, ranibizumab completely blocked both migration and proliferation induced by VEGF-A plus VEGF-B. Both VEGF-B variants did also not affect barrier function or claudin-1 expression in a normal or high-glucose environment. Accordingly, binding VEGF-A was enough to normalize a reduced TER and reinstate claudin-1 lost during treatment with this factor in combination with VEGF-B.

**Conclusions:**

Important properties and functions of REC seem not to be affected by any VEGF-B variant and targeting the key factor VEGF-A is sufficient to normalize growth factor-disturbed cells of this type.

**Electronic supplementary material:**

The online version of this article (doi:10.1007/s00417-015-2944-z) contains supplementary material, which is available to authorized users.

## Introduction

Members of the vascular endothelial growth factor (VEGF) family differently act upon retinal endothelial cells (REC): splice variants of VEGF-A, (VEGF-A_121_ and VEGF-A_165_) and of placenta growth factor (P*l*GF-1 and P*l*GF-2) stimulated proliferation of primary bovine REC (BREC) and immortalized BREC (iBREC), but only VEGF-A_165_ stimulated their migration and elevated their permeability [1–7]. Increased permeability of iBREC and human REC (HREC) monolayers induced by long-term exposure to VEGF-A_165_ correlated with loss of the tight junction (TJ) protein claudin-1, which became undetectable in the plasma membrane [3, 5, 8]. VEGF-B, another distinct member of the VEGF family, is expressed in the two differently spliced and proteolytically processed variants VEGF-B_167_ and VEGF-B_186_ [9–11]. Both variants are expressed in various normal tissues including ocular structures with VEGF-B_167_ typically being the dominant form, whereas an increased and predominant expression of VEGF-B_186_ was measured in malignant tumors [12]. Their 150 C-terminal amino acids are identical but they have distinct N-terminal ends resulting in different association with components of the extracellular matrix which bind VEGF-B_167_ , but not VEGF-B_186_ [10]. Similar to VEGF-A, both VEGF-B variants bind to VEGF receptor 1 (VEGFR1) and the non-tyrosine kinase receptor neuropilin (NRP), but in contrast to VEGF-A, they are not able additionally to activate VEGFR2 [11, 13]. Concerning the potential physiological functions of VEGF-B, contradictory results have been reported: there is some evidence that VEGF-B can act as a protective factor ensuring the survival of vascular endothelial cells and pericytes. For different cell types and experimental settings, VEGF-B was found to be either pro-angiogenic or anti-angiogenic [14]. VEGF-B is expressed by cells in the adult murine choroid, primary BREC, and pericytes [15, 16]. Although lack of VEGF-B did not affect the development of retinal vasculature under normal conditions, inhibition of VEGF-B resulted in decreased laser-induced choroidal neovascularization and ischemia-caused retinal neovascularization [15, 17]. If VEGF-B_167_ can stimulate proliferation or migration of (retinal) EC was investigated in vitro, but the results were not coherent [15, 18, 19]. Potential effects of VEGF-B on vascular permeability are also discussed controversially: overexpression of VEGF-B_186_ in the murine choroid resulted in choroidal neovascularization associated with an increased permeability, but similar effects were not observed in the murine brain [14, 20, 21]. In pursuit to further clarify VEGF-B’s physiological function it was observed that at least the splice variant VEGF-B_186_ is neuroprotective [21].

Pathogenesis and further development of diabetic retinopathy (DR) is associated with de-regulated expression of members of the VEGF-family: elevated levels of VEGF-A in the vitreous fluid were observed at all stages of DR, and P*l*GF was found up-regulated after transition to the proliferative form [22, 23]. Aqueous humor levels of P*l*GF were also significantly increased in eyes of patients with proliferative DR and diabetic macular edema (DME) [24]. VEGF-B was detected in the vitreous fluids of non-diabetic individuals at very low concentrations which were only slightly elevated in patients with DR [25]. Suppression of the detrimental effects of VEGF-A with the VEGF-binding proteins ranibizumab or aflibercept is a promising strategy to treat DR or DME which is likely caused by elevated permeability of REC [26–29]. In vitro, binding of VEGF-A by ranibizumab was sufficient to restore or prevent completely the VEGF-A-induced disturbance of the iBREC barrier or migration of these cells, even in the presence of other growth factors when surplus proliferation was only partly blocked [3, 5–7, 30]. Complete inhibition of iBREC proliferation stimulated by VEGF-A, P*l*GF, or a combination of both was achieved with aflibercept which can bind VEGF-A, VEGF-B, and P*l*GF [31–33]. However, aflibercept and ranibizumab both efficiently restored a functional iBREC barrier after extended treatment with complex growth factor mixes containing VEGF-A and P*l*GF [7].

To evaluate the potential effects of VEGF-B on important REC properties and functions, we studied whether presence of the variants VEGF-B_167_ or VEGF-B_186_, as single agents or in concert with VEGF-A_165_, affected proliferation or migration rates. In addition, changes of transendothelial resistance of an iBREC monolayer and of the amount or localization of the TJ-protein claudin-1 as markers for a functional REC barrier were measured during extended treatment with the above-mentioned growth factors. In view of a postulated anti-angiogenic effect of VEGF-B, improvement of the barrier function by counteracting VEGF-A was considered a possible outcome. As a question of potential therapeutic relevance, it was also investigated if binding VEGF-A with ranibizumab was sufficient to revert the effects induced by combinations of VEGF-A and VEGF-B variants or whether aflibercept was superior due to additional targeting of VEGF-B.

## Materials and methods

### Reagents, antibodies, and media

Recombinant human growth factors rhVEGF-A_165_ (SF21-expressed), rhVEGF-B_167_ (*E. coli*-expressed), and recombinant murine rmVEGF-B_186_ (SF21-expressed) were purchased from R&D Systems (Wiesbaden, Germany). The F(ab) fragment ranibizumab (10 mg/ml; Lucentis) of a humanized VEGF-A-binding antibody was a gift from Novartis Pharma GmbH (Nuremberg, Germany) [30]. The recombinant protein aflibercept (40 mg/ml; Eylea) consists of the VEGF binding domain 2 of VEGFR1, the binding domain 3 of VEGFR2, and an IgG-Fc part and was purchased from Bayer Health Care (Leverkusen, Germany) [32]. The humanized anti-VEGF-A antibody bevacizumab (25 mg/ml; Avastin from Roche Pharma, Grenzach-Wyhlen, Germany) was repackaged at the pharmacy of the University Hospital Ulm and provided in syringes which were stored at 4 °C [34]. In the laboratory, portions of the antibody were stored for less than 4 weeks in inert plastic vials. The CD20-specific humanized monoclonal antibody rituximab (10 mg/ml; MabThera) was purchased from Roche Pharma, and aliquot parts were stored in inert plastic vials at 4 °C [35]. Rabbit polyclonal antibodies binding to human claudin-1 (JAY.8) and detection antibodies for fluorescence microscopy (F(ab)_2_ conjugated with AlexaFluor 594) were from Life Technologies (Karlsruhe, Germany), and horseradish peroxidase-conjugated detection antibodies directed against rabbit or mouse IgG from BioRad (Munich, Germany).

### Cultivation of iBREC

Telomerase-immortalized microvascular endothelial cells from bovine retina (iBREC) were established and characterized in our laboratory [2]. The amount of human telomerase reverse transcriptase measured in iBREC was similar to that of the bovine homologue expressed by shortly cultivated primary BREC and did not result in significant changes of the phenotype: more than 99 % of the iBREC in a typical culture express von Willebrand factor, vascular endothelial cadherin, TJ-proteins claudin-1, claudin-3, claudin-5, and ZO-1, as well as VEGF receptors VEGFR1, VEGFR2, and neuropilin-1 which was confirmed every few weeks during prolonged cultivation. In addition, maintenance of cobblestone morphology was monitored every other day by microscopy. Although of bovine origin, iBREC can be stimulated with human growth factors, and reproducible responses were also considered indicative of a stable and authentic cell system ([2, 3, 5–7]; summarized in supplementary Table 1). iBREC were cultivated in Endothelial Cell Growth Medium MV (ECGM; Promocell, Heidelberg, Germany) containing 1 g/l glucose, 0.4 % Endothelial Cell Growth Supplement/H (ECGS/H, 90 μg/ml Heparin), 10 ng/ml epidermal growth factor, 103 nM hydrocortisone, and 5 % fetal calf serum (FCS) on fibronectin-coated (50 μg/ml; BD Biosciences, Corning, Amsterdam, the Netherlands) surfaces as previously described [2, 5]. Cells were used in the experiments at passages 20 to 40 counting from the stage of primary culture, for which stable expression of relevant proteins and reproducible response to growth factors had been confirmed. After cultivation for 3 days the confluent iBREC monolayer had formed a tight barrier indicated by a stable transendothelial resistance of ~50 Ohm × cm^2^ which is similar to values reported for monolayers of primary BREC or HREC [8, 36].

### Cell proliferation assay

After cultivation in serum-free medium (SFM; containing 0.4 % ECGS/H, 1 μg/ml fibronectin and 103 nM hydrocortisone) for 24 h, iBREC were exposed for 48 h to growth factors VEGF-A_165_, VEGF-B_167_, or VEGF-B_186_ as single agents or in combination, in the presence or absence of 100 μg/ml ranibizumab or 250 μg/ml aflibercept in SFM. Enzymatic conversion of WST-1 (Roche Diagnostics, Mannheim, Germany), indicative of proliferating cells, was determined as described [3, 31]. Values were normalized in relation to those obtained with control cells not treated with effectors. Results were shown only of experiments in which iBREC were treated with growth factors for 48 h because different effects were not observed during shorter or prolonged exposure.

### Cell migration assay

Transmembrane cell migration assays were performed in a modified Boyden chamber consisting of 12-well cell culture plates and inserts with a porous membrane (pore size 8.0 μm, ∅1 cm; Falcon, Corning) as previously described [3]. Their lower compartments were filled with SFM (without ECGS/H, but containing 5 μg/ml fibronectin) which was supplemented with VEGF-B_167_ or VEGF-B_186_ or these factors in combination with VEGF-A_165_ (25 μg/ml final concentration of each) with or without ranibizumab (6 or 60 μg/ml) or aflibercept (15 or 150 μg/ml). Inserted membranes were initially incubated for 1 h at 37 °C before 400 μl SFM (without ECGS/H and fibronectin) and a suspension of 10^5^ iBREC in 100 μl of this medium were added subsequently to the upper compartment. After 20 h at 37 °C, migration of cells through the pores of the membrane was assessed as described [3]. Values were normalized in relation to those obtained with control cells not treated with effectors.

### Treatment of iBREC with growth factors or inhibitors and subsequent analyses by Western blot and immunofluorescence staining

Prior to experiments with confluent iBREC, ECGM was replaced with serum-reduced medium (SRM, containing 0.4 % ECGS/H, 0.25 % FCS, 1 μg/ml fibronectin and 103 nM hydrocortisone) for 24 h. Cells were incubated for up to 2 days with single growth factors (10 to 100 ng/ml) or combinations (each growth factor at 50 ng/ml) before cell extracts were prepared. To investigate the potential of VEGF-binding proteins in restoring the barrier function of confluent iBREC, cells in SRM were pretreated with VEGF-A_165_ in combination with VEGF-B_167_ or VEGF-B_186_ (each growth factor at 50 ng/ml) for 30 h. Then the medium was changed to SRM containing the growth factors together with 0.1 to 100 μg/ml ranibizumab (≈2 to 2,000 nM), 0.25 to 250 μg/ml aflibercept (≈2 to 2,000 nM), 250 μg/ml bevacizumab, or rituximab (≈2 μM) before cell extracts were prepared 24 h later [5, 31]. Western blot analyses of whole cell extracts were performed as described [5]. After exposure of confluent monolayers of iBREC on fibronectin-coated, two-chamber slides with effectors, cells were fixed and claudin-1 visualized by immunofluorescence staining as described [3].

### Measurement of transendothelial electrical resistance

To assess paracellular permeability of iBREC, transendothelial electrical resistance (TER) was measured as described previously with minor modifications using polyethylenterephthalate membrane inserts (0.3 cm^2^, pore size 0.4 μm; Costar, Corning) coated overnight with 50 μg/ml fibronectin at 4 °C [5, 7]. Confluent iBREC monolayers formed after 3 to 4 days of cultivation were treated as described above and TER was measured at different time points (3, 6, 24, 30, 48, 52, and 72 h) after the addition of effectors. To avoid temperature-induced changes in TER, plates were kept on a warm plate at 37 °C during measurements. Normalized TER values were calculated in relation to the TER measured immediately before the medium was replaced by fresh medium containing the effectors.

### General considerations and statistical analyses

In all experiments, control cells were processed identically in medium only lacking the effector(s) under investigation. All experiments were repeated several times and in each experiment data were generated from multiple replicates. The Mann-Whitney U test was used to compare sets of experimental data and differences resulting in p-values below 0.05 were considered significant. Results were presented as conventional box-whiskers diagrams showing means and percentiles (75, 25 %).

## Results

### Ranibizumab efficiently blocked VEGF-B_167_- and VEGF-B_186_-induced proliferation and migration of iBREC

**Fig. 1:**
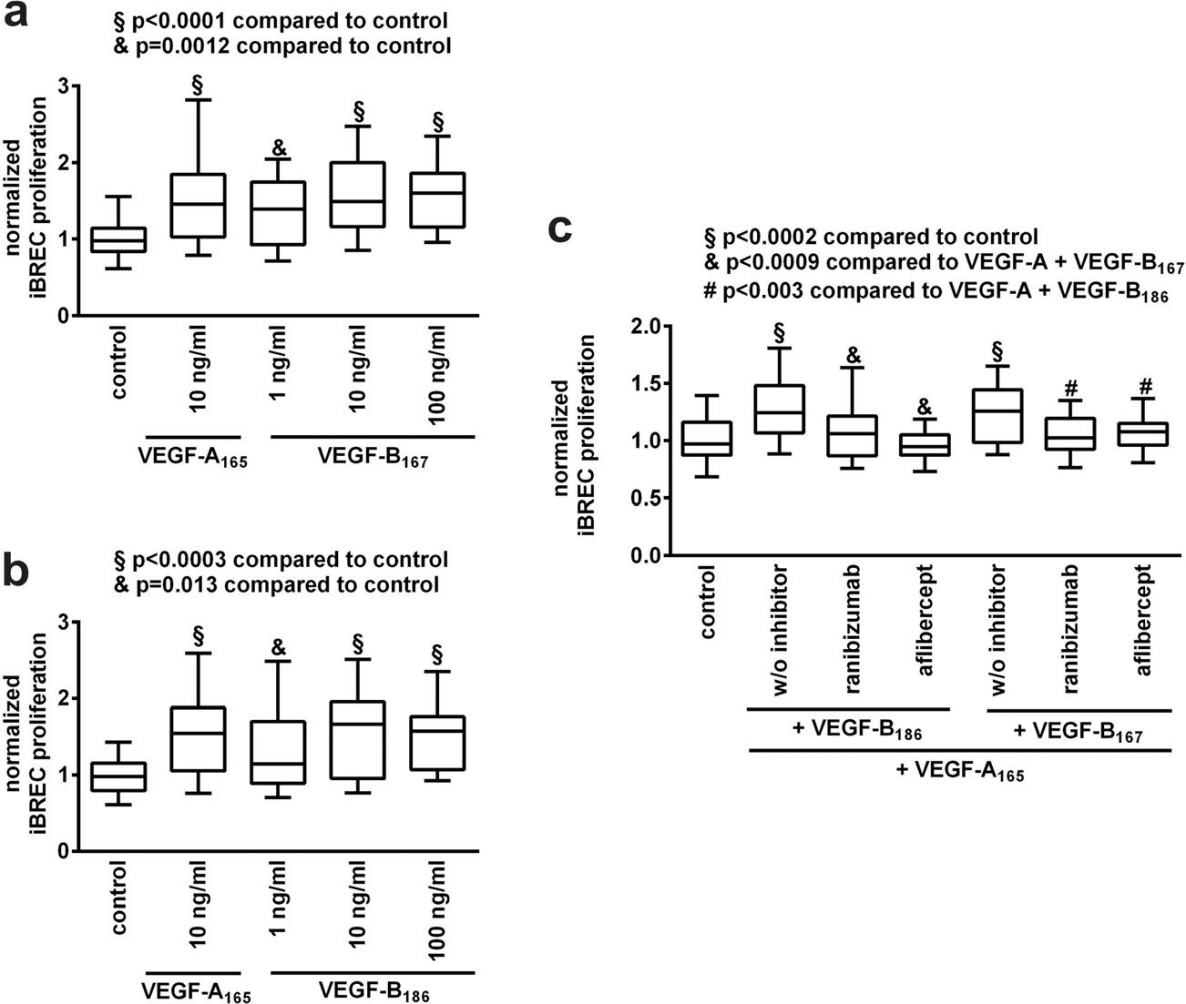
Proliferation of iBREC stimulated by VEGF-B_167_ or VEGF-B_186_ together with VEGF-A_165_ was completely blocked by ranibizumab. Serum-starved cells were stimulated with VEGF-B_167_ **(a)** or VEGF-B_186_ **(b)** for 48 h before conversion of WST-1 was determined as a measure of proliferation. **(c)** iBREC were treated with VEGF-A_165_ together with VEGF-B variants (10 ng/ml each) with or without 100 μg/ml ranibizumab or 250 μg/ml aflibercept (~2 μM of each inhibitor) for 48 h and proliferation of cells was assessed as described above. Values (*n* = 32) were normalized in relation to untreated cells. Proliferation of iBREC exposed to ≥ 1 ng/ml VEGF-B was significantly enhanced. Binding of VEGF-A was sufficient to completely inhibit stimulation of proliferation when both VEGF-A and VEGF-B were present

Because an involvement of VEGF-B splice variants in angiogenesis had been suggested, their effect on iBREC proliferation was studied: serum-starved cells were exposed to 10 ng/ml VEGF-A_165_, 1 to 100 ng/ml VEGF-B_167_ or VEGF-B_186_ for 2 days before conversion of WST-1 was determined as a measure of cell proliferation. Both VEGF-B isoforms enhanced proliferation of iBREC like VEGF-A_165_, confirming their induction of receptor-mediated signal transduction in these cells (Fig. 1a, b). However, when VEGF-A_165_ was present, they did not in any way modulate its effect on iBREC proliferation. Serum-starved cells were also incubated with growth factors as described above in the presence of clinically relevant concentrations of aflibercept or ranibizumab. Disproving the assumption that additional binding of VEGF-B by aflibercept might result in superior inhibition of iBREC proliferation stimulated with VEGF-A_165_ in combination with VEGF-B_167/186_, both VEGF-binding proteins were similarly efficient (Fig. 1c).

Both VEGF-B variants did not affect iBREC migration and also did not modulate its stimulation by VEGF-A_165_ (Table 1). In accordance with previous results showing that VEGF-A-induced migration of iBREC was specifically blocked by 6 or 60 μg/ml ranibizumab (≈0.13 or 1.3 μM) or 15 μg/ml aflibercept (≈0.13 μM), both inhibitors applied at these concentrations lowered migration back to basal levels despite VEGF-B variants being present as co-factors (Table 1) [3, 31]. High concentrations of aflibercept (150 μg/ml ≈ 1.3 μM) resulted in unspecific overcompensation below the level of basal migration as earlier reported [31].

**Table 1:** Migration of iBREC stimulated with growth factor combinations including VEGF-A and VEGF-B was completely inhibited by ranibizumab

VEGF-A_165_	VEGF-B_167_	VEGF-B_186_	Ranibizumab		Aflibercept		Normalized migration rate [mean + SD]	Comparison GF(s)^a^ to control	Comparison GF(s)^a^ to GF(s) with ranibizumab	Comparison GF(s)^a^ to GF(s) with aflibercept
			60 μg/ml	6 μg/ml	150 μg/ml	15 μg/ml				
–	–	–	–	–	–	–	1.00 ± 0.30	–	–	–
x	–	–	–	–	–	–	1.44 ± 0.56	*p* < 0.008	–	–
–	x	–	–	–	–	–	0.94 ± 0.45	*p* > 0.05	–	–
–	–	x	–	–	–	–	0.78 ± 0.43	*p* > 0.05	–	–
x	x	–	–	–	–	–	1.65 ± 0.45	*p* < 0.0001	–	–
x	x	–	x		–	–	1.23 ± 0.44	*p* > 0.05	*p* = 0.001	–
x	x	–	–	x	–	–	1.21 ± 0.34	*p* > 0.05	*p* = 0.0001	–
x	x	–	–	–	x	–	0.32 ± 0.33	*p* < 0.0001	–	*p* < 0.0001
x	x	–	–	–	–	x	1.24 ± 0.52	*p* > 0.05	–	*p* = 0.0007
x	–	x	–	–	–	–	1.34 ± 0.16	*p* < 0.0001	–	–
x	–	x	x	–	–	–	1.05 ± 0.16	*p* > 0.05	*p* < 0.0001	–
x	–	x	–	x	–	–	1.09 ± 0.16	*p* > 0.05	*p* < 0.0001	–
x	–	x	–	–	x	–	0.28 ± 0.20	*p* < 0.0001	–	*p* < 0.0001
x	–	x	–	–	–	x	0.82 ± 0.30	*p* > 0.05	–	*p* < 0.0001

Migration of serum-starved iBREC during 24 h against VEGF-A_165_, VEGF-B_167_ or VEGF-B_186_ (25 ng/ml each) or combinations of these factors was measured in a modified Boyden-Chamber assay with or without 100 μg/ml ranibizumab or 250 μg/ml aflibercept. Values (*n* = 36) were normalized in relation to those obtained with control cells not treated with effectors

^a^ *GF* growth factor

### VEGF-B_167_ and VEGF-B_186_ did not affect iBREC barrier function

**Fig. 2:**
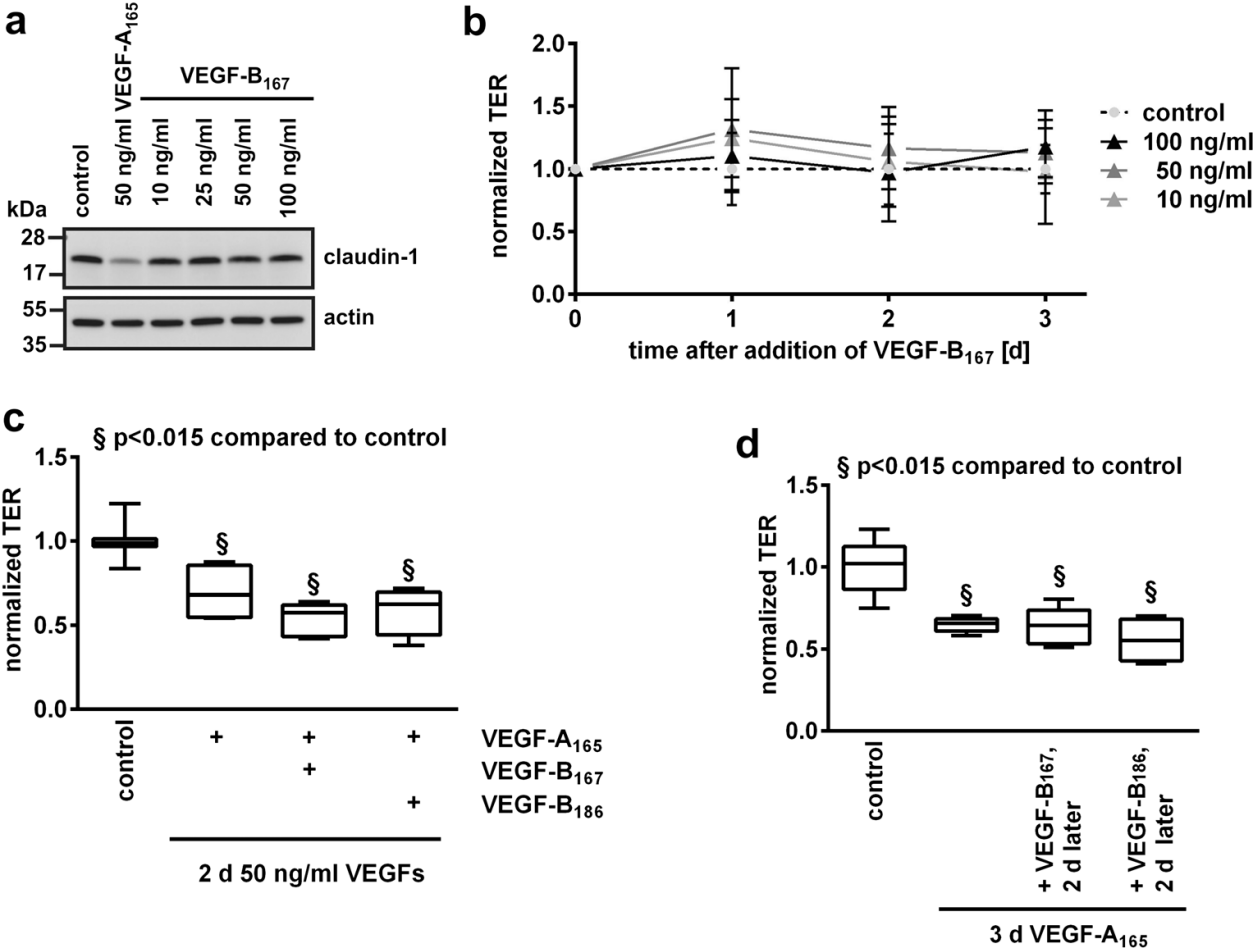
VEGF-B_167_ or VEGF-B_186_ did neither affect TER or claudin-1 expression nor modulate VEGF-A-induced barrier disturbances. **(a, b)** iBREC were exposed for up to 3 days to 10 to 100 ng/ml VEGF-B_167_ before cell extracts were prepared to determine claudin-1 by Western blot **(a)** or TER was measured at indicated time points **(b)**. Claudin-1 expression was only lower in the presence of VEGF-A_165_ , whereas VEGF-B_167_ variants did not affect expression of this TJ protein or directly measured TER. Similar results were obtained with VEGF-B_186_. **(c, d)** iBREC were incubated with VEGF-A_165_ together with either VEGF-B_167_ or VEGF-B_186_ **(c)** or the cells were pretreated with VEGF-A_165_ for 2 days before VEGF-B_167_ or VEGF-B_186_ (50 ng/ml each) were added **(d)**. TER was measured 24 h later. The VEGF-A_165_-caused TER decrease was neither prevented nor reverted by any VEGF-B splice variant

The barrier function of iBREC was assessed by measuring TER of confluent cells. This approach is non-invasive and has the distinct advantage that the same culture can be monitored easily during long-term experiments by multiple subsequent measurements. In addition, presence of TJ-protein claudin-1, a cell surface marker indicating a functional barrier, was monitored [5, 7]. Because changes occasionally observed early after addition of growth factors were considered less relevant, we focused on barrier disturbance established in the cultures during cultivation for more than 24 h. iBREC were treated with 10 to 100 ng/ml VEGF-B for up to 3 days before cell extracts were prepared for Western blot analyses. TER was measured over the same period at different time points. As shown in Fig. 2a, claudin-1 had disappeared after treatment with VEGF-A_165_ , but amounts were not altered even after extended treatment with VEGF-B_167_ or VEGF-B_186_ (Fig. 2a). We confirmed that localization of claudin-1 was not affected under these conditions (data not shown), since particularly the quantity of plasma membrane-localized claudin-1 was shown to correlate strongly with TER [3, 5]. Accordingly, significantly changed TER values were not observed (Fig. 2b).

### VEGF-B_167_ and VEGF-B_186_ did not modulate the effect of VEGF-A_165_ on iBREC barrier function

Although both VEGF-B splice variants did not affect the barrier function of iBREC, their possible enhancing or counteracting the action of the most important effector VEGF-A_165_ remained to be ruled out. Therefore, iBREC were incubated with VEGF-A_165_ together with either VEGF-B_167_ or VEGF-B_186_ (50 ng/ml each) for 48 h before TER was measured or cell extracts were prepared. A similar loss of claudin-1 and reduction of TER was observed with all combinations tested, indicating that both splice variants of VEGF-B did not modulate the strong effect of VEGF-A_165_ on the iBREC barrier (Fig. 2c). When the iBREC barrier had already been disrupted with VEGF-A_165_, a normalizing effect was also not observed during subsequent treatment with VEGF-B_167_ or VEGF-B_186_ (50 ng/ml each) for additional 24 h (Fig. 2d).

To mimic hyperglycemia in diabetes patients, the influence of elevated glucose levels on the actions of the different growth factors was also studied: iBREC were cultivated for 3 days in medium containing 3 g/l (≈17 mM) D-glucose instead of the normal 1 g/l (≈5.6 mM) D-glucose before VEGF-A_165_ and VEGF-B were added. Claudin-1 was determined 1 day later by Western blot, and its presence was not affected by the glucose concentration in the culture medium. Likewise, loss of this TJ-protein as a consequence of treatment with VEGF-A_165_ alone or together with VEGF-B was completely independent of the amount of glucose in the medium (data not shown).

### Inactivating VEGF-A_165_ was sufficient to reverse iBREC barrier dysfunction induced by treatment with this factor in combination with VEGF-B_167_ or VEGF-B_186_

**Fig. 3:**
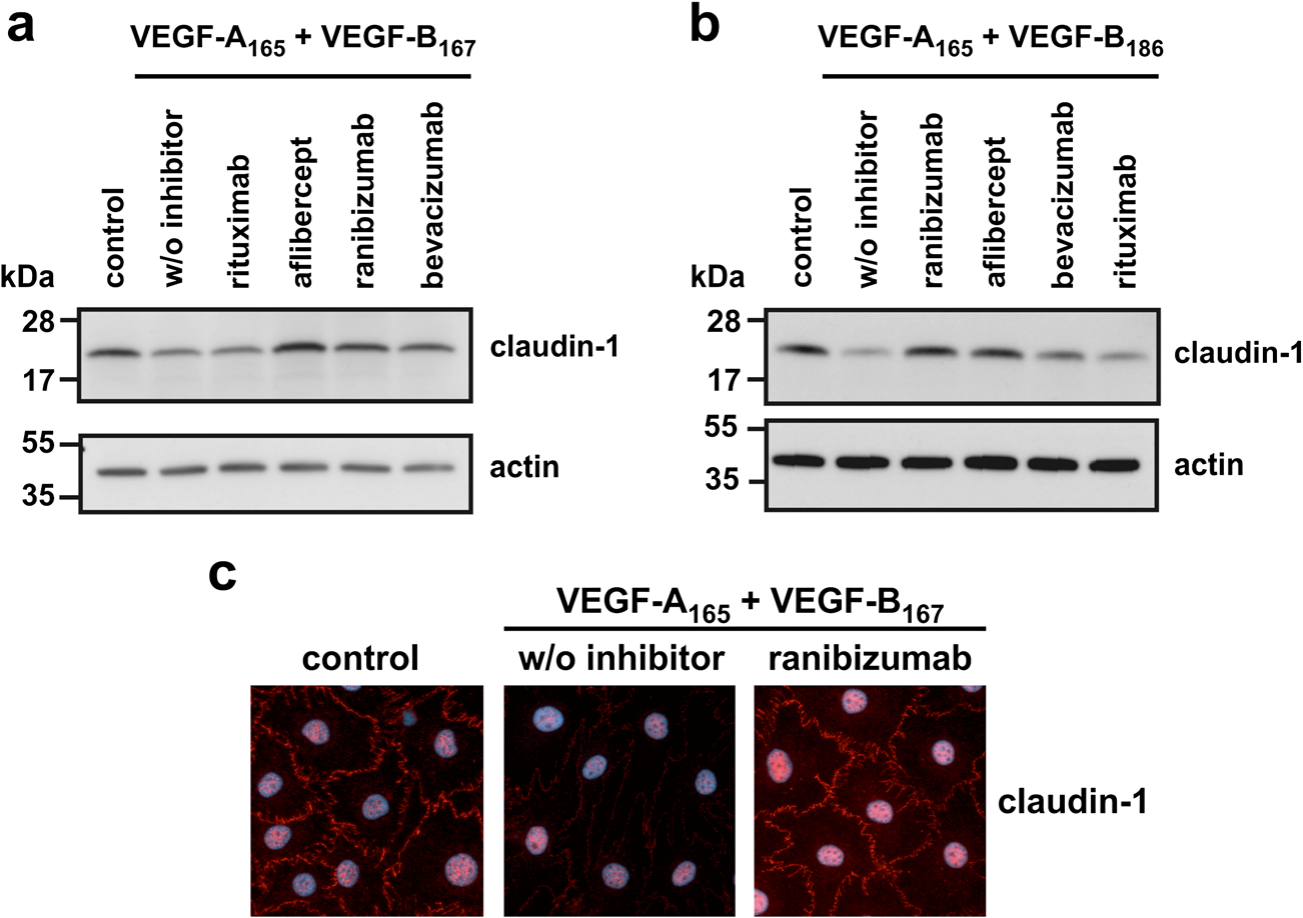
Binding of VEGF-A_165_ was sufficient to reinstate lost claudin-1 in the presence of VEGF-B **(a, b)** iBREC were treated with combinations of growth factors (each at 50 ng/ml) for 30 h before 100 μg/ml ranibizumab or 250 μg/ml aflibercept, bevacizumab or rituximab were added. Cell extracts were prepared 24 h later and analyzed by Western blot. Treatment with any of the VEGF-A-binding proteins, but not with the control antibody rituximab, resulted in reappearance of lost claudin-1. **(c)** iBREC were treated with VEGF-A_165_ together with VEGF-B_167_ for 30 h before 100 μg/ml ranibizumab was added for 24 h. The TJ protein claudin-1 was then visualized by immunofluorescence staining. Claudin-1 vanished from the plasma membrane during growth factor treatment, but normal staining was seen after subsequent incubation with the VEGF-A-binding protein

Because both VEGF-B variants seemed not to contribute to the disturbance of the iBREC barrier we assumed that binding of VEGF-A_165_ by ranibizumab should be sufficient to reverse the effect of VEGF-A_165_ even in the presence of VEGF-B_167_ or VEGF-B_186_. To test this hypothesis, confluent iBREC were exposed to various combinations of VEGF-A_165_, VEGF-B_167_ and VEGF-B_186_ (50 ng/ml each) for 30 h and then treated with different VEGF-binding proteins at clinically relevant concentrations for an additional 24 h before cellular extracts were prepared. As a specificity control, the potential of the chimeric antibody rituximab to revert VEGF-A induced changes was also tested [35]. This antibody does not bind to any protein in iBREC [31]. Ranibizumab and bevacizumab, both binding only VEGF-A, and aflibercept targeting both VEGF-A and VEGF-B all similarly re-established normal claudin-1 presence in iBREC, but rituximab did not have any effect (Fig. 1a, b). As expected, the amount of plasma membrane bound claudin-1 was dramatically reduced in iBREC treated with VEGF-A_165_ and VEGF-B_167_ (50 ng/ml each), but it reappeared when cells were exposed to ranibizumab at a clinically relevant concentration of 100 μg/ml (Fig. 3c). Similar results were obtained for iBREC treated with VEGF-A_165_ together with VEGF-B_186_ (data not shown).

**Fig. 4:**
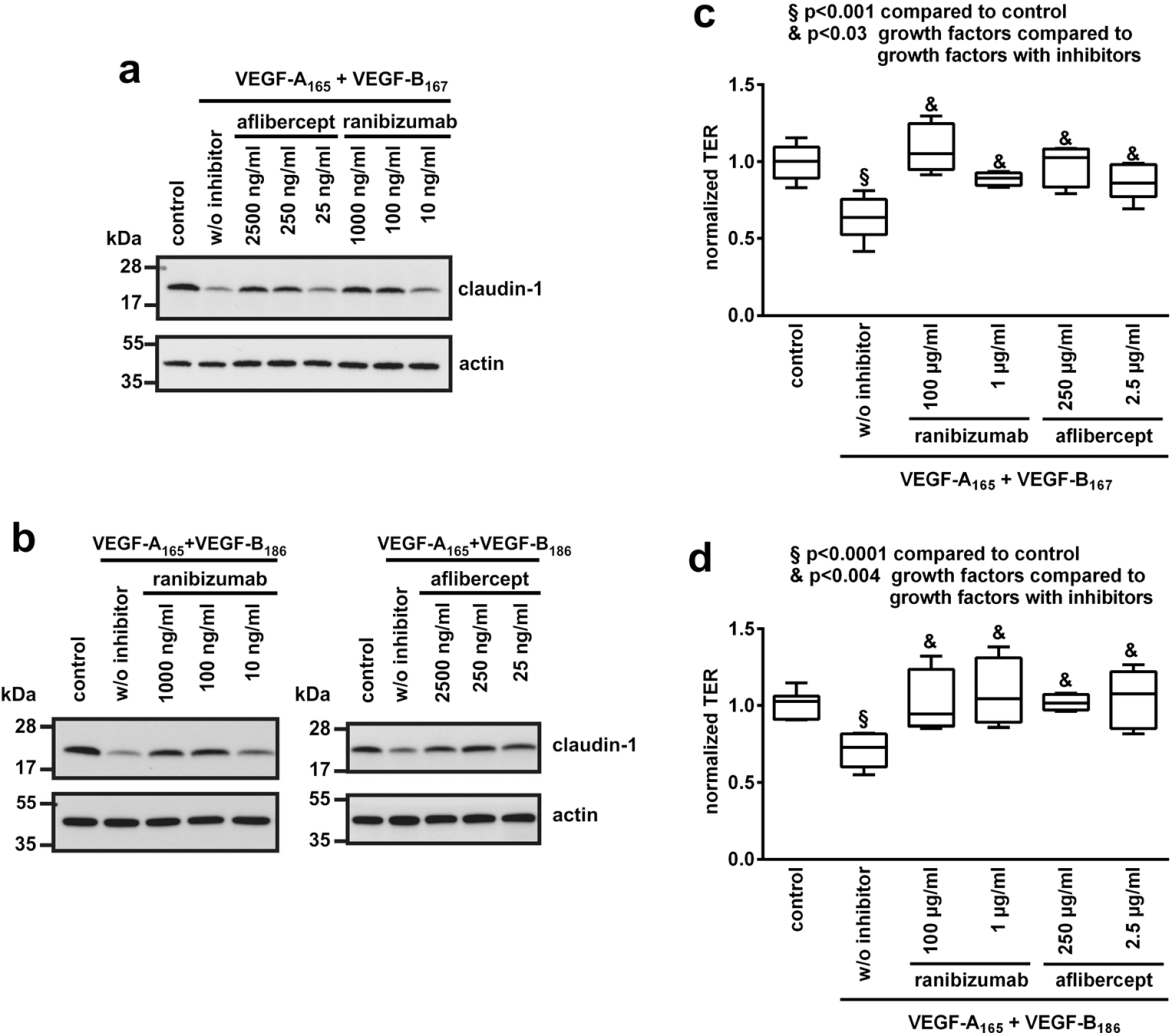
Aflibercept and ranibizumab reinstated lost claudin-1 with similar efficacy **(a,b)** iBREC were treated with VEGF-A_165_ + VEGF-B_167_ **(a)** or VEGF-A_165_ + VEGF-B_186_ **(b)** for 30 h before ranibizumab or aflibercept were added for another 24 h. To neutralize 50 ng/ml (≈1 μM) of the VEGF variants, inhibitors were used at various concentrations in the range of ≈0.01 to 1 μM corresponding to 10 to 1,000 ng/ml ranibizumab or 25 to 2,500 ng/ml aflibercept. **(c, d)** Ranibizumab or aflibercept were added to iBREC pretreated for 30 h with VEGF-A_165_ together with VEGB_167_ **(c)** or VEGF-B_186_ **(d)** as described above and TER was measured 24 h later. Normalizing effects of the VEGF-binding proteins on induced disturbances of the iBREC barrier were similar

To compare their relative efficacies, various amounts of the VEGF-binding proteins ranibizumab and aflibercept were added to iBREC pretreated for 30 h with VEGF-A_165_ in combination with VEGF-B_167_ or VEGF-B_186_ (50 ng/ml each, ≈ 1 μM). Cell extracts were prepared 24 h later, and expression of claudin-1 was analyzed by Western blot (Fig. 4a, b). Lost claudin-1 was completely brought back by 1 μg/ml ranibizumab or 2.5 μg/ml aflibercept (≈2 μM), concentrations even 100× lower than values typically achieved after intraocular injection. Also sufficient were 0.1 μg/ml ranibizumab or 0.25 μg/ml aflibercept (≈200 nM), although VEGF-A_165_ cannot be bound completely by the inhibitors under these conditions: About 5 ng/ml free VEGF-A_165_ remained in the culture supernatant as determined by ELISA. This amount is obviously too low to affect strongly the expression of claudin-1 by iBREC. When VEGF-A_165_ was present at 10-fold excess over the VEGF-binding proteins, loss of claudin-1 could not be reverted indicating an insufficient inhibition.

We also measured directly whether ranibizumab and aflibercept can normalize the iBREC barrier disturbed by VEGF-A in the presence of the potentially co-acting VEGF-B: when ranibizumab or aflibercept were added to iBREC which had been treated for 30 h with VEGF-A_165_ in combination with VEGF-B_167_ or VEGF-B_186_ (50 ng/ml each), the substantially reduced TER normalized in all cases within 24 h. To achieve normalization of TER, even much lower concentrations of the inhibitors than those reached after intra vitreal injection were found to be sufficient (Fig. 4c, d).

## Discussion

Targeting members of the VEGF family is a promising option in the therapy of diabetic macular edema [27–29]. Whereas the different functions of VEGF-A have been intensively studied, the role of VEGF-B still appears rather diffuse [12–21]. In the normal eye, VEGF-B_167_ is the predominantly expressed variant whereas substantial amounts of VEGF-B_186_ seem to be present only in malignant tumors [12]. Because retinal endothelial cells form an important part of the blood-retina-barrier, a functional impact of VEGF-B on this cell type might be highly relevant to therapeutic concepts. Therefore, we investigated whether the two main variants VEGF-B_167_ and VEGF-B_186_ affected the barrier properties of iBREC or modulated the changes induced by the key factor VEGF-A_165_. Since migration and proliferation of REC are involved in retinal neovascularization as a characteristic of proliferative DR, potential stimulation of these processes by VEGF-B variants was also investigated.

VEGF-B_167_ and VEGF-B_186_ indeed enhanced proliferation of iBREC after exposure for 2 days which is in accordance with other observations [19]. In contrast, proliferation of primary HREC was not stimulated by VEGF-B_167_ after treatment for only 24 h [18]. In addition to the shorter time of exposure, weaker stimulation of cell proliferation might have been due to conditions associated with cultivating primary REC, e.g. presence of inhibiting antibiotics and other cell types like pericytes. It can be concluded from their effect on proliferation that the used recombinant human and murine VEGF-B variants activate signal transduction in bovine REC. Like P*l*GF, another member of the VEGF family, the studied VEGF-B variants did not stimulate migration of iBREC which may be a consequence of their binding to VEGFR1 without co-activating VEGFR2 [6, 37]. Interestingly, ranibizumab completely blocked both iBREC migration and proliferation when these processes had been stimulated by VEGF-A together with VEGF-B. Additional inhibition of VEGF-B, as achieved with aflibercept, was obviously not necessary to normalize proliferation, indicating that in the presence of the dominant VEGF-A, parallel activation of independent signalling pathways is not relevant to the overall effect. It could be speculated that VEGF-A expression is somehow induced by VEGF-B, but neither did VEGF-A binding ranibizumab block proliferation stimulated only by VEGF-B nor could VEGF-A be detected in these experiments (data not shown). A possible, but unconfirmed mechanism underlying the complete inhibition of proliferation by ranibizumab is its binding and inactivation of putative VEGF-A/VEGF-B heterodimers which have been described [9]. If, instead of VEGF-B, P*l*GF acts as a co-stimulator, binding of both P*l*GF and VEGF-A is necessary to block proliferation completely, emphasizing the distinct properties of the related factors [6]. The three VEGF family members VEGF-A, VEGF-B, and P*l*GF all bind to the Ig domain 2 of VEGFR1, but whereas VEGF-A and P*l*GF interact with the same region of VEGFR1, differing contact points between the receptor and VEGF-B have been identified [38]. This may be the reason for different subsequent signal transduction and the substantially lower affinity of VEGF-B compared to VEGF-A, which in turn provides a simple explanation of our observation that iBREC proliferation induced by VEGF-A was not notably affected by equimolar amounts of VEGF-B. That VEGF-B, like P*l*GF, failed to initiate basal or alter VEGF-A-stimulated migration supports our previously made assumption that iBREC migration is almost exclusively mediated through VEGFR2 together with NRP [6].

The barrier function of REC was also not affected by the VEGF-B variants, which was clearly indicated by a constant TER and stable claudin-1 expression over several days of incubation. This is in accordance with the previous observation that vascular permeability was not affected by VEGF-B_167_ [14]. This was also confirmed by overexpressing VEGF-B_167_ in the murine choroid by means of an adenoviral vector. In contrast VEGF-B_186_ induced permeability in this experimental context [20]. Retinal and choroidal endothelial cells might respond differently to VEGF-B, but it seems more likely that this is an effect only of very high amounts of the growth factor which do not reflect physiological conditions [39]. Interestingly, the factors VEGF-B_167/186_ and P*l*GF-1/-2, which do not influence permeability of iBREC monolayers, all bind and activate VEGFR1, but not VEGFR2, supporting our previously made assumption that disturbance of the REC barrier is mainly mediated through VEGFR2 with VEGF-A being its most important ligand [7]. Both variants of VEGF-B also did not modulate the strong effect of VEGF-A_165_ on the TJ-protein claudin-1 and TER. Loss of claudin-1 or reduction of TER induced by VEGF-A_165_ was neither prevented nor more pronounced even when VEGF-B_167_ or VEGF-B_186_ were added subsequently, or when cells were kept under glucose stress. Therefore, the assumption that binding of VEGF-A is sufficient to normalize a barrier function disrupted by VEGF-A_165_ in combination with VEGF-B_167_ or VEGF-B_186_ seemed reasonable and could be confirmed. Even at concentrations well below values achieved after intraocular injection, ranibizumab completely reversed reduction of TER and reinstated lost claudin-1 in iBREC exposed to combinations of these growth factors. The efficacy of aflibercept was similar, but not better, indicating that its additional binding of VEGF-B is not relevant to the normalization of the REC barrier.

Our results support the assumption that neither VEGF-B_167_ nor VEGF-B_186_ can play a significant role in the control of permeability of the REC barrier even when long-term exposure as a consequence of pathogenic processes in the eye has to be considered. In addition, marked effects of VEGF-B_167_ or VEGF-B_186_ on REC proliferation and migration, the hallmarks of angiogenic processes, were also not recognized. Accordingly, normalization of a REC barrier disrupted by VEGF-A in the presence of VEGF-B was achieved by targeting the key factor VEGF-A without additional inactivation of VEGF-B being an advantage or disadvantage and the same conclusion can be drawn for blocking proliferation or migration in an environment containing both VEGF-A and VEGF-B. Besides the primary therapeutic effects of VEGF inhibitors with a broader binding specificity, their interference with neuroprotection potentially provided by VEGF-B may also play a role in some cases and should be taken into account [21].

## Electronic supplementary material

Below is the link to the electronic supplementary material.

Supplementary Table S1 (DOCX 14 kb)
