# Supplementary material for: Binding of VEGF-A is sufficient to abrogate the disturbing effects of VEGF-B together with VEGF-A on retinal endothelial cells
Source: Graefes Arch Clin Exp Ophthalmol. 2015 Feb 8;253(6):885–94. doi: 10.1007/s00417-015-2944-z (PMC4445774; doi:10.1007/s00417-015-2944-z)
Supplement: Supplementary file 1 — (DOCX 14 kb) [file 417_2015_2944_MOESM1_ESM.docx]

Supplementary Table 1: Similar properties of primary and immortalized BREC

|  |  | BREC | |
| --- | --- | --- | --- |
|  |  | primary | immortalized |
|  |  |  | (iBREC) |
| General |  |  |  |
| morphology |  | “cobble stone” | “cobble stone” |
| pericytes in culture |  | ca. 5% | none |
| contact inhibition |  | yes | yes |
| barrier stability (TER) |  | 50 Ω x cm² | 50 Ω x cm² |
| uptake of acetylated LDL |  | yes | yes |
|  |  |  |  |
| Expression of proteins |  |  |  |
| proteins typical for EC: |  | yes | yes |
| von Willebrand factor |  | yes | yes |
| vascular endothelial cadherin |  | yes | yes |
| TJ-proteins: |  |  |  |
| claudin-1 |  | yes | yes |
| claudin-5 |  | yes | yes |
| occludin |  | yes | yes |
| VEGF receptors: |  |  |  |
| VEGFR1 |  | yes | yes |
| VEGFR2 |  | yes | yes |
| NRP |  | yes | yes |
| protein typical for retinal pericytes: |  |  |  |
| αSMA |  | no | no |
|  |  |  |  |
| Stimulation of proliferation by |  |  |  |
| VEGF-A_165_ |  | yes | yes |
| VEGF-A_121_ |  | yes | yes |
| P*l*GF-1 |  | yes | yes |
| P*l*GF-2 |  | yes | yes |
| VEGF-B_167_ |  | not done | yes |
| VEGF-B_186_ |  | not done | yes |
|  |  |  |  |
| Stimulation of migration by |  |  |  |
| VEGF-A_165_ |  | yes | yes |
| VEGF-A_121_ |  | not done | no |
| P*l*GF-1 |  | no | no |
| P*l*GF-2 |  | not done | no |
| VEGF-B_167_ |  | not done | no |
| VEGF-B_186_ |  | not done | no |
|  |  |  |  |
| Induction of barrier dysfunction  (TER measurement) |  |  |  |
| VEGF-A_165_ |  | yes, strongly | yes, strongly |
| VEGF-A_121_ |  | not done | yes, weakly |
| P*l*GF-1 |  | no | no |
| P*l*GF-2 |  | no | no |
| VEGF-B_167_ |  | not done | no |
| VEGF-B_186_ |  | not done | no |
|  |  |  |  |
| VEGF-A induced loss of claudin-1 |  | not done | yes, strongly |
| VEGF-A induced loss of occludin |  | yes, strongly | yes, strongly |
